# Short high‐fat diet interferes with the physiological maturation of the late adolescent mouse heart

**DOI:** 10.14814/phy2.14474

**Published:** 2020-07-08

**Authors:** Heidi Hynynen, Maija Mutikainen, Nikolay Naumenko, Anastasia Shakirzyanova, Tomi Tuomainen, Pasi Tavi

**Affiliations:** ^1^ A.I. Virtanen Institute for Molecular Sciences University of Eastern Finland Kuopio Finland

**Keywords:** Akt, calcium signaling, echocardiogram, energy metabolism, PI3K, transcription

## Abstract

Dietary fats are essential for cardiac function. The metabolites of fats known as fatty acids provide most of the energy for cardiac tissue, serve as building blocks for membranes and regulate important signaling cascades. Despite their importance, excess fat intake can cause cardiac dysfunction. The detrimental effects of high‐fat diet (HFD) on cardiac health are widely investigated in long‐term studies but the short‐term effects of fats have not been thoroughly studied. To elucidate the near‐term effects of a HFD on the growth and maturation of late adolescent heart we subjected 11‐week‐old mice to an 8‐week long HFD (42% of calories from fat, 42% from carbohydrate, *n* = 8) or chow diet (12% of calories from fat, 66% from carbohydrate, *n* = 7) and assessed their effects on the heart in vivo and in vitro. Our results showed that excessive fat feeding interferes with normal maturation of the heart indicated by the lack of increase in dimensions, volume, and stroke volume of the left ventricles of mice on high fat diet that were evident in mice on chow diet. In addition, differences in regional strain during the contraction cycle between mice on HFD and chow diet were seen. These changes were associated with reduced activity of the growth promoting PI3K‐Akt1 signaling cascade and moderate changes in glucose metabolism without changes in calcium signaling. This study suggests that even a short period of HFD during late adolescence hinders cardiac maturation and causes physiological changes that may have an impact on the cardiac health in adulthood.

## Graphical abstract

This study suggests that even a short period of high fat intake during late adolescence hinders maturation of the heart and causes physiological changes that may have an impact on the cardiac health in adulthood.

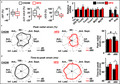

## 1 INTRODUCTION

Fatty acids (FAs), the metabolites of dietary fats, are used for energy, cell and organelle membrane building, and signaling pathway regulation throughout the body. Although essential, excess consumption of FAs is known to cause adverse systemic effects through oxidative stress, inflammation, and insulin resistance (Chalkiadaki & Guarente, 2012; Furukawa et al., 2004; Xu et al., 2003), making the balanced intake of FAs essential.

Like many organs the heart is vulnerable to excess FA consumption. Although cardiac tissue is efficient in using FAs as energy, a diet high in fat induces serious cardiac complications (Stanley, Recchia, & Lopaschuk, 2005). Thus excessive fat intake associated with increasing prevalence of obesity poses a population‐wide risk to cardiac health (Golay & Bobbioni, 1997; Pi‐Sunyer, 2009). Especially adolescent obesity is a predisposing factor to cardiac pathologies later in life as recent extensive long‐term follow‐up studies show that high body mass index in late adolescence increases the risk of cardiomyopathy, hypertension, arrhythmias, arterial disease, and heart failure alongside with increased mortality associated with these diseases in adulthood in humans (Robertson et al., 2019; Twig et al., 2017). In mice long‐term high‐fat diet (HFD) consumed in juvenility induces contractile dysfunction and hypertrophy, as well as functional and structural changes in the heart resembling those seen in diabetic cardiomyopathy (Abdurrachim et al., 2014; Calligaris et al., 2013; Fang et al., 2008; Wang, Li, Zhao, Peng, & Zuo, 2015; Zeng, Vaka, He, Booz, & Chen, 2015). From the developmental perspective, late adolescence is an interesting period because although the heart is nearly fully developed at young age, significant developmental steps still take place before adulthood. Pre‐ and postnatal heart consumes mainly glucose, but during maturation the main energy substrate shifts toward FAs (Stanley et al., 2005). At the same time, the heart undergoes the final phase of structural maturation characterized by an increase in left ventricle mass and volume associated with an augmentation of stroke volume and cardiac output (Wiesmann et al., 2000). Thus, it is evident that deviations from normal conditions, for example obesity and excessive dietary fat intake can interfere with the final maturation steps of the heart.

Although a long‐term HFD is known to be detrimental to the maturing heart, the effects of a short‐term HFD are less known. To investigate the primary effects of a HFD on the heart at this developmental stage, we subjected 11‐week‐old mice to a short 8‐week HFD. Organ‐level changes were studied by measuring the functional parameters and dimensions of the left ventricle with echocardiography and speckle tracking strain analysis *in vivo*. Cellular level changes were elucidated by examining energy metabolism, as well as calcium and growth signaling pathways *in vitro*.

## 2 MATERIALS AND METHODS

### 2.1 Animals

Male C57Bl/6JOlaHsd (Harlan Laboratories Inc.) mice were housed in standard conditions and under authorization from the Lab Animal Centre of the University of Eastern Finland. Animal studies were approved by the National Animal Experiment Board of Finland and carried out following the guidelines of the Finnish Act on Animal Experimentation and Directive 2010/63/EU of the European Parliament. Starting at 11 weeks of age, mice were fed either a high‐fat Western diet containing 42% of calories from fat and 42% from carbohydrate (TD.88137, Envigo) or standard a chow diet containing 12% of calories from fat and 66% from carbohydrates (2016S, Envigo). Animals were sacrificed after 8 weeks, at 19 weeks of age. The total number of mice was 32, with 16 on the chow diet and 16 on the HFD. Assay‐specific number of animals is stated in the corresponding figure legend.

### 2.2 In vivo cardiac imaging

**Figure 1:**
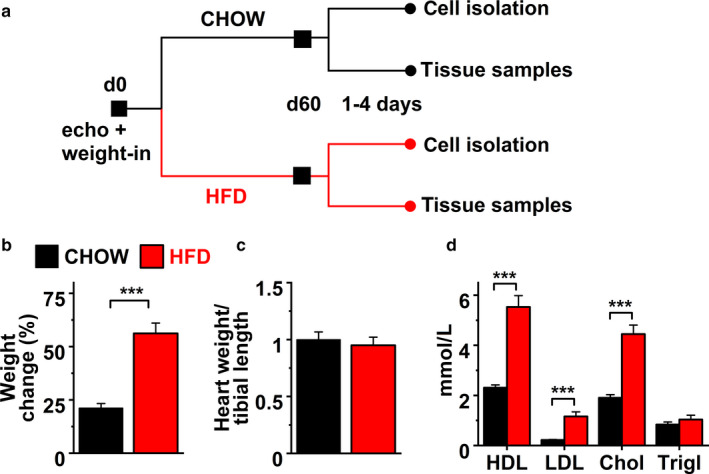
(a) Flow chart of the study design. Black rectangles indicate echocardiography and weigh‐in, and circles indicate the day of sacrification. CHOW indicates chow diet; HFD, high‐fat diet. (b) Weight change during the diet (chow *n* = 7, HFD *n* = 8), (c) heart weight to tibial bone length ratio at sacrification (*n* = 4) and (d) values of different lipid species measured from the plasma collected at sacrification (*n* = 4). Unpaired Student´s *t* test: ***P < .001

Echocardiographic imaging and the weigh‐in of the mice were done the day before the diet began (d0) and 1–4 days before the study endpoint (d60; Figure 1a). Echocardiographic measurements were performed as described by Mutikainen *et al*. with Vevo2100 Imaging System (FUJIFILM VisualSonics Inc) assuming cardiac symmetry from 2D M‐mode (Mutikainen et al., 2016). Cardiac parameters in Table 1 were analyzed from parasternal short axis M‐mode measurements in which the papillary muscles serve as an anatomic landmark of the mid‐ventricular level. The analysis was conducted from consecutive cardiac cycles, including three systolic and four diastolic phases. In addition to the conventional analysis of the left ventricle, three consecutive cardiac cycles were acquired and analyzed with speckle tracking from mid‐ventricular B‐mode short‐axis view for radial strain and time‐to‐peak strain measurements (VevoStrain, FUJIFILM VisualSonics Inc.). The endocardium was tracked with 48 points, dividing the left ventricle into six segments. “Peak strain” and “time‐to‐peak strain” were analyzed in each segment, where “strain” is defined as change in length during the contraction and relaxation of myocardium. Therefore, strain reflects the amount of stretching of the myocardial tissue whereas strain rate indicates the speed of the deformation. Data smoothing for polar plots of segmental data was performed with OriginLab2019 using the Adjacent‐Averaging method with a points of window value of 5 (OriginLab).

**Table 1:** Measurements of body weight and echocardiographic parameters before diet (d0) and 8 weeks later (d60). Change percent (d60−d0)/d0 × 100

	Chow diet (*n* = 7)			High fat diet (*n* = 8)		
	d0	d60	Change %	d0	d60	Change %
Body weight (g)	23.9 ± 0.6	29.1 ± 1.3^†††^	20.9 ± 2.2	24.9 ± 0.7	38.9 ± 1.6^†††^	56.2 ± 4.8^***^
LV Mass (mg)	129.5 ± 10.1	159.1 ± 11.4^†††^	23.4 ± 2.5	128.6 ± 11.4	131.5 ± 7.7	7.0 ± 9.5
Diastolic LVAW (mm)	0.8 ± 0.04	0.89 ± 0.04^†^	8.9 ± 2.7	0.8 ± 0.04	0.85 ± 0.02	7.6 ± 5.4
Systolic LVAW (mm)	1.1 ± 0.1	1.2 ± 0.06^††^	11.9 ± 2.7	1.1 ± 0.04	1.14 ± 0.03	3.7 ± 4.5
Diastolic LVID (mm)	4.1 ± 0.1	4.4 ± 0.06^†^	7.2 ± 2.4	4.17 ± 0.07	4.08 ± 0.09	−2.2 ± 1.7^**^
Systolic LVID (mm)	3.1 ± 0.2	3.21 ± 0.1	4.8 ± 3.6	3.15 ± 0.1	3.07 ± 0.09	−2.0 ± 3.5
Diastolic LVPW (mm)	0.8 ± 0.04	0.9 ± 0.03	6.9 ± 3.0	0.8 ± 0.06	0.84 ± 0.02	7.9 ± 8.7
Systolic LVPW (mm)	1.1 ± 0.1	1.2 ± 0.05^†^	10.5 ± 3.6	1.12 ± 0.04	1.12 ± 0.03	0.9 ± 5.6
Diastolic LV Vol (µl)	74.1 ± 5.1	86.1 ± 3.1^†^	18.5 ± 6.4	77.6 ± 3.1	73.8 ± 3.7	−4.7 ± 3.9**
Systolic LV Vol (µl)	38.6 ± 4.6	41.6 ± 3.3	13.8 ± 9.6	39.9 ± 3.2	37.4 ± 2.8	−3.4 ± 8.3
LV stroke vol (µl)	35.6 ± 2.1	44.5 ± 2.5^††^	26.0 ± 6.2	37.7 ± 2.4	36.4 ± 2.1	−1.1 ± 8.1^*^
Fractional shortening (mm)	0.99 ± 0.1	1.15 ± 0.07^†^	17 ± 6.2	1.02 ± 0.07	1.01 ± 0.05	2.6 ± 9.4
HR (bpm)	425.7 ± 16.4	469.1 ± 13.7^†^	10.8 ± 3.6	413.5 ± 6.7	441.4 ± 19.2	6.7 ± 4
Cardiac output (ml/min)	15.1 ± 0.9	20.9 ± 1.3^††^	39.5 ± 8.1	15.6 ± 0.9	15.9 ± 0.7	4.3 ± 7**
Ejection fraction (%)	48.9 ± 3.3	51.8 ± 2.9	7.2 ± 5.4	48.9 ± 3.1	49.5 ± 2.1	4.0 ± 7.8

Abbreviations: AW, anterior wall; d, diastolic; HR, heart rate; ID, internal diameter; LV, Left ventricular; PW, posterior wall; s, systolic; Vol, volume.

^†††^ *P* < .001, ^††^ *P* < .01, ^†^ *P* < .05 between start and end of the diet of each animal (paired Student’s *t* test), ***P < .001, **P < .01, *P < .05 percentual change between diets (unpaired Student’s *t* test).

### 2.3 Quantitative PCR

Total RNA was extracted from heart tissue with TRI Reagent (Merck KGaA). 1 µg of RNA was taken into cDNA synthesis using RevertAid First Strand cDNA Synthesis Kit (ThermoFisher Scientific) performed according to the manufacturer's protocols. Quantitative PCR was performed with specific fluorescent primers and probes (Table S1) using TaqMan Thermo master mix (ThermoFisher Scientific) with StepOnePlus device (ThermoFisher Scientific). Ribosomal 18S expression was used in normalization and fold change calculated compared to chow diet.

### 2.4 Western Blotting

A piece of cardiac tissue was homogenized in lysis buffer (50 mM Tris‐HCl pH7.5, 150 mM NaCl, 1 mM EDTA, 1% Triton‐X‐100, 0.5% Na‐deoxy cholate, 0.1% SDS, 10% glycerol) supplemented with Protease Inhibitors Cocktail (Roche), 50 mM NaF and 1 mM Na_3_VO_4_. Lysates were centrifuged in 3000 ***g***, 10 min, 4°C, and supernatant was collected. Protein concentrations were measured using Protein dye assay reagent (BioRad). Equal amounts of protein samples were loaded on SDS‐PAGE gel and transferred to nitrocellulose membrane. Proteins were immunoblotted with primary antibodies (PI3K p110α sc7174, Santa Cruz Biotechnology Dallas, TX, USA; Akt1 07‐416, pAkt1 (Ser473) 04‐736, PI3K p85 06‐195, Merck KGaA) and fluorescent Cy‐labeled or HRP‐conjugated secondary antibodies, and detected with GelDoc imager (Bio‐Rad). Quantification of proteins was done with Image Lab™ Software (Bio‐Rad) normalizing to Ponceau labeled total protein and fold change calculated compared to chow diet.

### 2.5 Blood analysis

Circulating lipid levels were measured from plasma. Blood samples were collected at the time of sacrifice into 0.5 M EDTA containing tubes and centrifuged 10 min at 2,000×***g*** to separate the plasma. Lipid levels were measured using photometric methods at a commercial animal diagnostic laboratory (Movet Oy).

### 2.6 Cardiomyocyte isolation

Cardiomyocytes were obtained as described in AfCS Procedure Protocol ID PP00000 125 (http://www.signaling‐gateway.org/data/cgi‐bin/ProtocolFile.cgi?pid=PP00000125 *)* with some changes. Briefly, mice were injected with heparin 30 min before sacrification. The heart was cannulated and placed in a Langendorff perfusion apparatus and flushed for 4 min with perfusion buffer containing 113 mM NaCl, 4.7 mM KCl, 0.6 mM KH_2_PO_4_, 0.6 mM Na_2_HPO_4_, 1.2 mM MgSO_4_×7H_2_O, 0.032 mM Phenol Red, 20.5 mM NaHCO_3_, 10 mM KHCO_3_, 10 mM Hepes, 30 mM Taurine, 5.5 mM Glucose, 10 mM 2,3‐Butanedione monoxime (BDM). Sodium bicarbonate concentration in the perfusion buffer was adjusted to 20.5 mM from the original protocol's 12.5 mM in order to keep pH equal to 7.4 in 5% CO_2_ incubator. To enzymatically dissociate the cardiomyocytes, the heart was perfused for 7 min with perfusion buffer supplemented with 0.025 mg/mL Liberase (Roche) and 0.14 mg/mL Trypsin (Sigma‐Aldrich). The hearts were removed from the apparatus and the left ventricles were dissected in myocyte digestion buffer. The ventricles were further cut to small pieces, moved to a tube kept at +37°C water bath and mixed by pipetting with a fire polished Pasteur pipette. Deviating from the original protocol, the pieces were left to settle to the bottom of the tube, and floating dissociated cells were pipetted from the supernatant to a new tube containing perfusion buffer supplemented with 10% FBS and 12.5 µM CaCl_2_. Fresh digestion buffer (+37°C) was added to the pieces, mixed, left to settle. The dissociated cells were combined with the previously transferred cells in buffer with 10% FBS and 12.5 µM CaCl_2_. This step was repeated several times until the tissue pieces were fully dissociated. Dissociated cells were spun down and suspended to buffer containing 10% FBS and 62 µM CaCl_2_. Calcium reintroduction was continued stepwise (112, 212, 500 µM) until the final concentration of 1 mM.

### 2.7 Seahorse metabolism analysis

Aerobic and anaerobic energy metabolism of isolated cardiomyocytes was measured with Seahorse extracellular flux analyzer (Agilent Technologies). After calcium reintroduction, cardiomyocytes were spun down and suspended in XF assay medium (XF Base Medium minimal DMEM with 2 mM GlutaMAX [ThermoFisher Scientific] and 12 mM BDM [Sigma‐Aldrich]) containing either 4.5 g/L glucose (Sigma‐Aldrich) or 0.2 mM BSA‐conjugated palmitate (Sigma‐Aldrich). The cells with glucose containing medium were supplemented with equal amount of unconjugated BSA. Cells were inspected under a microscope, counted manually with a Bürker chamber, and a suspension containing 2000 rod shaped cells was plated on Matrigel (BD Matrigel matrix, growth reduced, BD Biosciences) coated XF24 cell culture microplates. The cells were let to attach for 1 hr at + 37°C 5% CO_2_. Four wells were left empty for background measurements. After incubation, wells were microscopically checked to ensure that cardiomyocytes were attached. The analyzer was prepared with a calibration plate containing XF Calibrant solution according to the manufacturer's instructions. This assay consisted of three basal metabolic rate measurements followed by the addition of FCCP (carbonyl cyanide‐4‐(trifluoromethoxy) phenylhydrazone, 1.5 µM), which induces maximal respiration, and 10 measurements of maximal metabolic rate. Background values were subtracted from raw measurements and results were normalized to total protein concentration measured using Protein dye assay reagent (BioRad).

### 2.8 Calcium imaging

Calcium signals were measured as described by Mutikainen et al. (2016) from isolated cardiomyocytes.

### 2.9 Statistical analyses

Data are presented as mean ± standard error of the mean (SEM). When comparing two groups or change percentages, statistical significance was evaluated using Student´s *t* test for unpaired samples. Follow‐up data for echocardiography between d0 and d60 for each individual mouse were analyzed with paired Student´s *t* test. *P* < .05 was considered statistically significant.

## 3 RESULTS

### 3.1 HFD affects body weight and plasma lipids

To investigate the early effects of HFD in adolescent mouse heart, 11‐week old mice were fed either normal chow diet (12% of calories from fat, 66% from carbohydrates, *n* = 7) or HFD (42% of calories from fat, 42% from carbohydrates, *n* = 8) for 8 weeks. Echocardiography before either diet showed no difference in structural or functional parameters of the left ventricles between the study groups (Table 1). During the course of the study, body mass increased significantly more in mice on HFD compared to chow diet (56.4% versus 21.1%, Figure 1b), but the ratio of the total heart weight to tibial bone length did not change between the groups (Figure 1c). As expected, HFD increased plasma cholesterol levels in HFD compared with chow diet (Figure 1d). However, plasma triglycerides were similar in mice on HFD compared to chow diet (Figure 1d), in line with the previous report (Sanchez et al., 2018).

### 3.2 HFD affects the structure and function of the late adolescent heart

**Figure 2:**
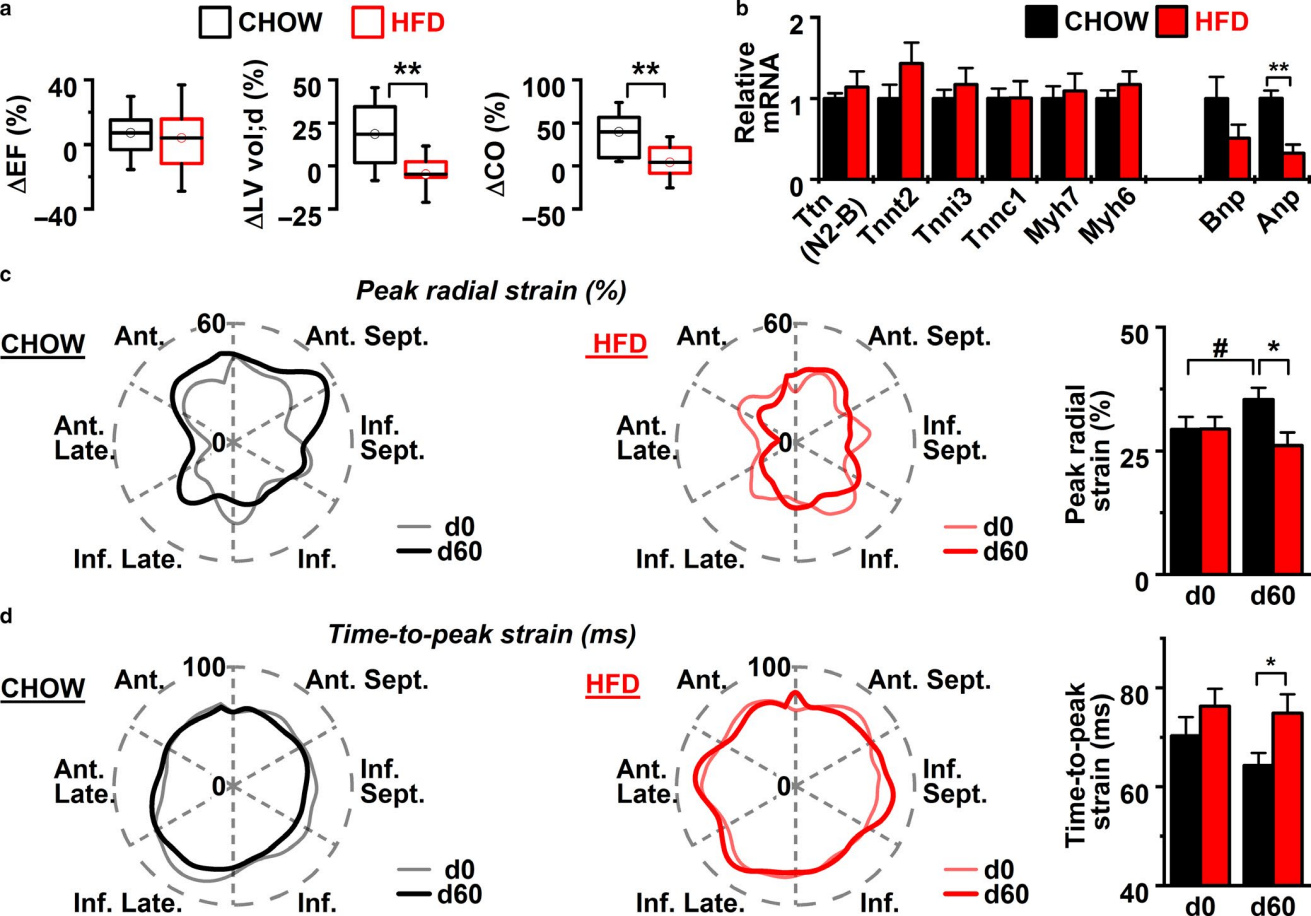
(a) The percentage value of change in diastolic ejection fraction (EF), left ventricular volume (ΔLV vol;d) and cardiac output (ΔCO) after 8 weeks (60 days) of chow or high‐fat diet (HFD) (Box—25%–75%, wiskers—1.5*SD*; chow *n* = 7, HFD *n* = 8). (b) Expression of cardiac contractile element genes (chow *n* = 5, HFD *n* = 4). (c) and (d) Segmental (left and middle) and average (right) (c) peak radial strain and (d) time‐to‐peak strain data of left ventricles of chow and HFD mice before the diet commencement (d0) and after 60 days on the diet (d60) (chow *n* = 7, HFD *n* = 8). Unpaired Student’s *t* test: **P < .01, *P < .05. Paired Student’s *t* test: #*P* < .05

According to echocardiography, the hearts of mice on chow diet underwent a period of growth and maturation from 11 to 19 weeks of age (Table 1). During these 8 weeks the left ventricular (LV) mass (estimate assuming cardiac symmetry from 2D M‐mode), systolic LV posterior wall thickness, diastolic and systolic LV anterior wall (LVAW) thickness, diastolic LV internal diameter (LVID) as well as diastolic LV volume, stroke volume, fractional shortening, heart rate (HR) and cardiac output (CO) were all significantly increased (Table 1). In contrast, cardiac parameters of HFD group did not change during this 8‐week period. As a result, when percentual change between d0 and d60 were determined, mice on HFD had significantly smaller change in diastolic LVID, diastolic LV volume, LV stroke volume, and CO compared to chow diet (Figure 2a, Table 1). These changes were not associated with changed cardiac contractile element gene expression (Figure 2b), but were accompanied with decreased expression of *Anp* (Figure 2b), a commonly used marker of cardiac hypertrophy (Tavi, Laine, Weckstrom, & Ruskoaho, 2001).

To assess the cardiac function in more detail, we used speckle tracking strain analysis (An et al., 2016; de Lucia et al., 2019), which is comparable to cardiac MRI, the golden standard in characterizing myocardial dynamics (Onishi et al., 2015; Pedrizzetti, Claus, Kilner, & Nagel, 2016). This analysis showed that the peak radial strain in chow diet had increased from d0 to d60, indicated by the growth in area depicting the myocardial stretching during the cardiac cycle (Figure 2c). This growth was absent in HFD group. In addition, time‐to‐peak strain, reflecting the speed of maximum strain development was shorter at the study end point in chow diet group compared to HFD group resulting in a significant difference between the dietary groups (Figure 2d). Since the time‐to‐peak strain depicts the length of the cardiac cycle the result suggests that the developmental increase in the speed of the contraction cycle is hindered by HFD diet.

### 3.3 Short‐term HFD does not affect cardiomyocyte calcium signaling

**Figure 3:**
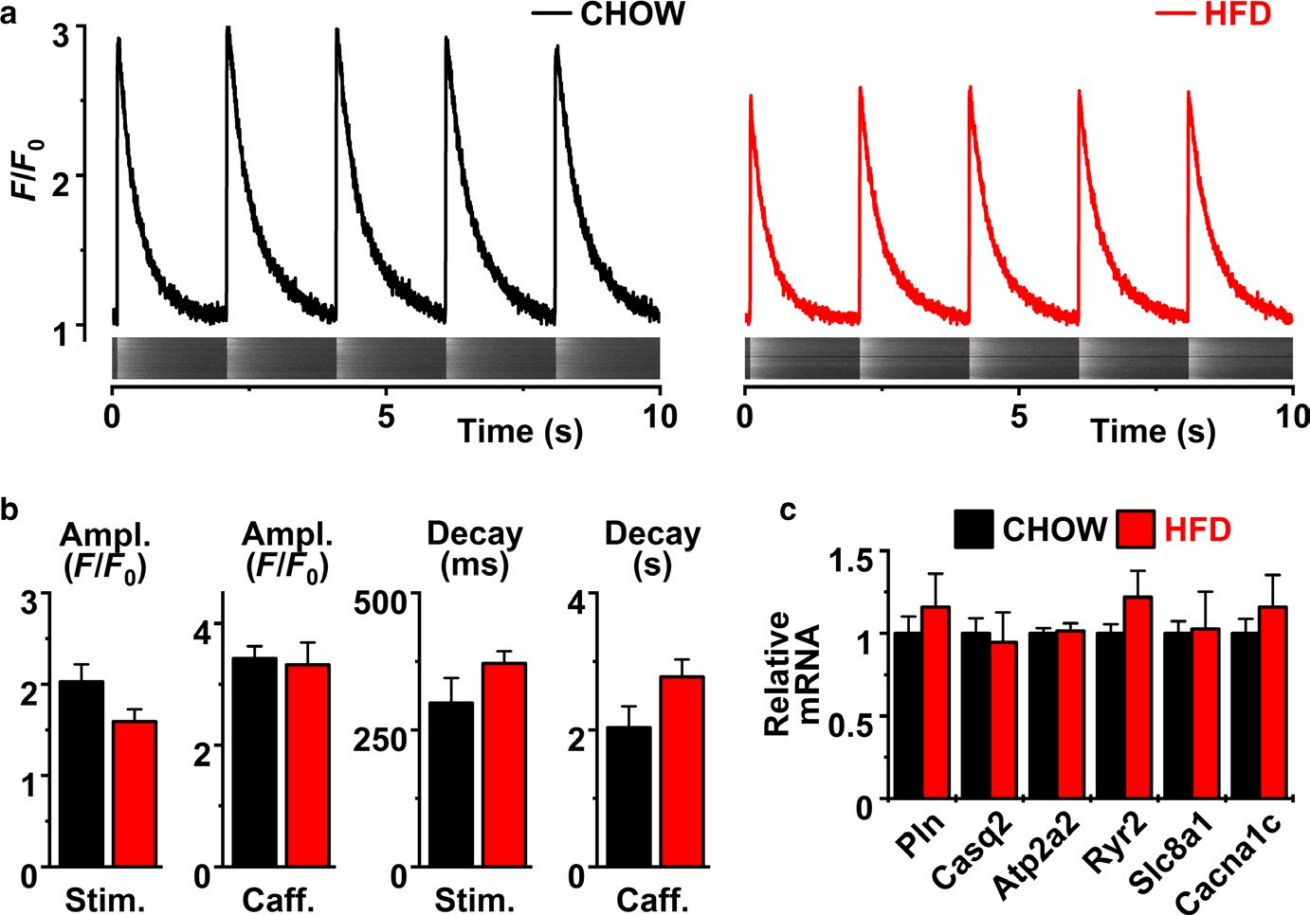
(a) Representative electrically triggered [Ca^2+^]_i_ transients from chow (left) and high fat diet (right) mice ventricular cardiomyocytes, (b) basal (Stim.) and caffeine (Caff.)‐induced calcium transient amplitudes (Ampl.) and decay time of basal (Stim.) and caffeine (Caff.)‐induced calcium transients (chow *n* = 6 cells, HFD *n* = 7 cells). (c) Expression of calcium handling‐related genes Pln, Phospholamban; Casq2, Calsequestrin; Ryr2, Ryanodine receptor 2 (chow *n* = 5, HFD *n* = 4)

Calcium transient amplitudes, SR‐Ca‐loading (caffeine induced amplitude) or decay showed no difference in chow and HFD mice (Figure 3b). Furthermore, the expressions of genes centrally involved in calcium signaling were not changed (Figure 3c). Combined these results suggest that HFD ‐induced changes in left ventricle function are not due to compromised calcium signaling of the myocytes.

### 3.4 HFD induces changes in energy metabolism in late adolescent mice hearts

**Figure 4:**
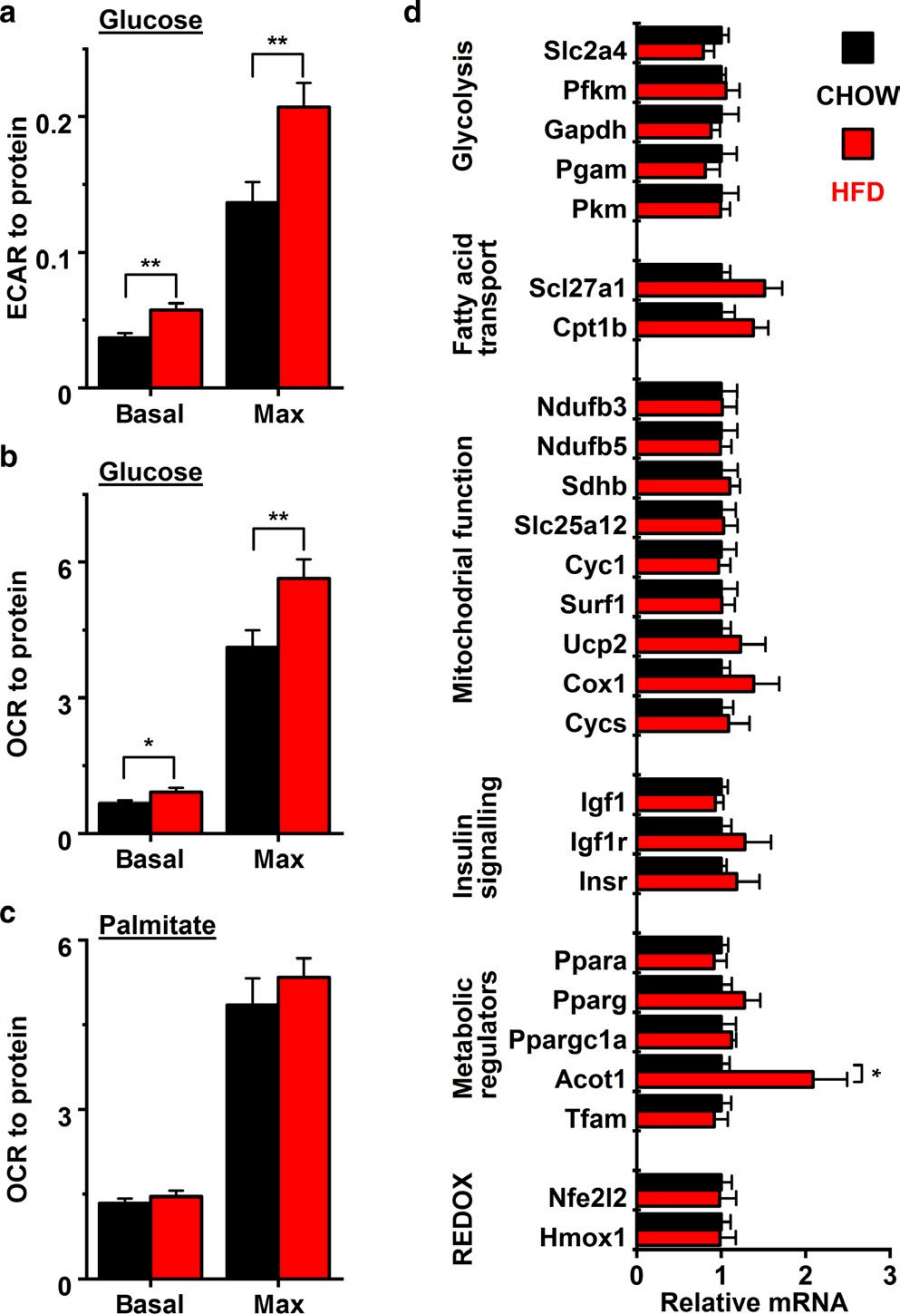
Quantification of (a) extracellular acidification rate (ECAR) (chow basal *n* = 36 wells, HFD basal *n* = 37 wells, chow max *n* = 34 wells, HFD max *n* = 36 wells) and (b) oxygen consumption rate (OCR) (chow *n* = 33 wells, HFD *n* = 40 wells) with glucose, and (c) oxygen consumption rate (OCR) with palmitate (chow *n* = 35 wells, HFD *n* = 40 wells) as substrate. (d) Expression of genes related to glycolysis, fatty acid transport, mitochondrial function, insulin signalling, metabolic regulation, and ROS signalling (chow *n* = 5, HFD *n* = 4). ***P < .001, **P < .01, *P < .05

When examining oxidative and anaerobic metabolism we discovered that when glucose was given as sole substrate, HFD led to higher basal and maximal metabolism. HFD increased both basal and maximal extracellular acidification rates (Figure 4a) as well as basal and maximal oxygen consumption rates (OCR; Figure 4b). Interestingly, these effects were seen only when glucose was given as a substrate whereas the utilization of palmitate was not different between dietary groups (Figure 4c).

HFD has been shown to interfere with energy metabolism by inducing mitochondrial and respiratory chain remodeling (Chen, Li, Zhang, Zhu, & Gao, 2018), reducing mitochondrial density and inducing mitochondrial damage (Dong, Li, Sreejayan, Nunn, & Ren, 2007) as well as downregulating the expression of regulators of FA oxidation, such as PGC‐1α and PPARα (Elezaby et al., 2015). Although analysis of cellular respiration did not indicate decline in mitochondrial function, we next wanted to see if changes would be detectable at transcriptional level. Therefore, we measured a number of transcripts involved in glycolysis, FA transport, mitochondrial function, insulin signaling, metabolic regulation and ROS signaling and found that the only gene that had changed expression was Acyl‐CoA thioesterase 1 (*Acot1*, Figure 4d) with increased expression in HFD hearts. These results suggest that a short‐term HFD does not cause drastic metabolic remodeling but induces a shift of energy metabolism to more glycolytic direction.

### 3.5 HFD inhibits PI3K‐Akt‐pathway

**Figure 5:**
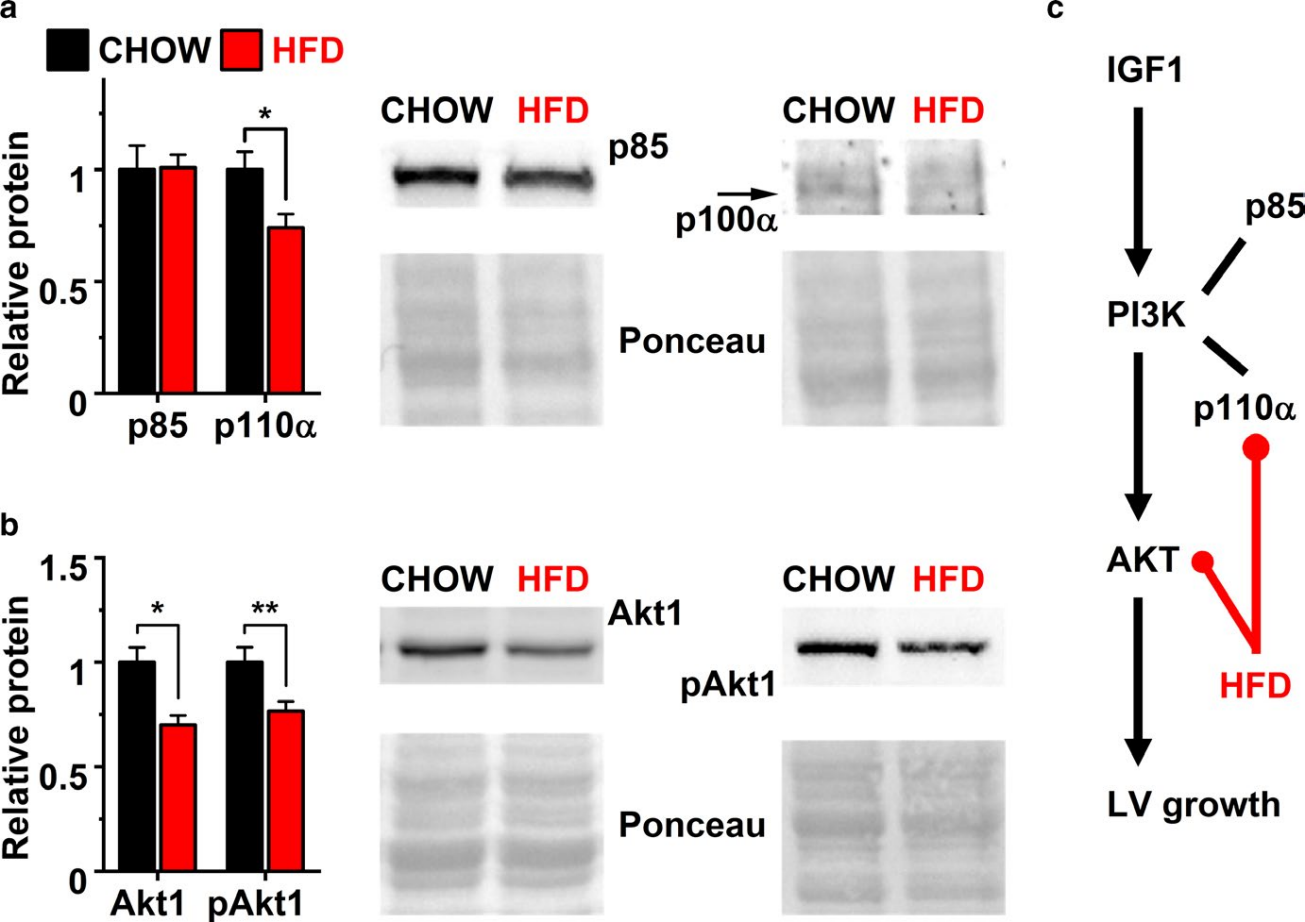
Relative protein expression of (a) PI3K subunit p85 and p110α (chow *n* = 8, HFD *n* = 7), (b) PI3K target Akt (*n* = 8) and its phosphorylated from (pAkt1) (*n* = 8). (c) Flow chart depicting inhibitory targets of high‐fat diet (red lines) in the PI3K‐Akt‐pathway (black lines). **P < .01, *P < .05

High‐fat diet has been shown to interfere with the InsR‐PI3K‐Akt‐pathway that regulates cardiac growth (DeBosch & Muslin, 2008; Ouwens et al., 2005; Shiojima et al., 2002). In our study, the expression of insulin receptor (*Insr*), insulin like growth factor (*Igf1*), and insulin like growth factor 1 receptor (*Igf1r*) were all unchanged (Figure 4d). However, the protein expression of p110α (Figure 5a), the catalytic subunit of phosphoinositide 3‐kinase (PI3K) and its target RAC‐alpha serine/threonine‐protein kinase (Akt1, also called PKB; Figure 5b) were both downregulated in HFD mice hearts. Also, the expression of phosphorylated Akt1 (pAkt1) was reduced by 23.3% in HFD group (Figure 5b) while the regulatory subunit of PI3K, p85 remained unaffected (Figure 5a). This suggest that the activity of PI3K‐Akt‐pathway was inhibited already by short term HFD (Figure 5c), which could explain the observed inhibition of LV maturation.

## 4 DISCUSSION

According to both animal and human studies, developing heart is particularly sensitive to high dietary fat content (Ayer, Charakida, Deanfield, & Celermajer, 2015; Calligaris et al., 2013) suggesting that high plasma FA levels can interfere with pathways promoting cardiac development. This study demonstrates that an 8‐week long HFD started at 11‐weeks of age hindered the normal physiological maturation of the heart. At the cellular level these changes were associated with changes in glucose metabolism and reduced activity of PI3K‐Akt1 signaling cascade.

During normal postnatal growth of mouse, the functional capacity of the heart is increased first by rapid growth of the heart mass, followed by more moderate growth period. Mice are considered mature adults after 12 weeks of age, but there are still changes and growth seen in the heart until 16 and even 29 weeks of age (Leu, Ehler, & Perriard, 2001; Wiesmann et al., 2000). Accordingly, between 11 and 19 weeks of age our control mice (chow diet) showed an increase in left ventricle mass, systolic and diastolic LVAW diameter, systolic posterior LV wall thickness, diastolic LV diameter and volume, stroke volume, fractional shortening, and HR resulting altogether in 39.5% augmentation of the CO (Table 1). In addition, speckle tracking based strain analysis revealed developmental increase in the peak radial strain of the LV in control mice during this 8‐week period (Figure 2c) indicating that the maturation of the heart was still on‐going. Interestingly, when cardiac parameters of HFD mice were compared before and after the diet, no change in any of the measured parameters was detected (Table 1). In fact, after 8 weeks of diet HFD mice had significantly smaller percentual change in diastolic LVID and volume, as well as smaller LV stroke volume and CO when compared to control animals on chow diet (Table 1). Furthermore, speckle tracking strain analysis showed that HFD mice had smaller peak radial strain and longer time‐to‐peak strain at the end of the diet, indicating that HFD interfered with the development of contractile force and speed of left ventricles. Overall, the structural and functional parameters of the HFD mice hearts did not indicate pathological changes such as hypertrophy or contractile dysfunction that are usually associated with HFD (Dong et al., 2007; Fang et al., 2008; Hua et al., 2013; Zhang et al., 2012). Instead, comparison of the LV function between HFD mice and their age‐matched controls on chow diet showed that HFD specifically suppressed physiological maturation of the heart during late adolescence.

In addition to restricted growth of left ventricle, our results show that HFD in late adolescence increased both basal and maximal aerobic and anaerobic utilization of glucose (Figure 4). Development of pathological cardiac hypertrophy and especially heart failure is associated with energy substrate switch from FAs to glucose (Tuomainen & Tavi, 2017). However, since the HFD mice hearts showed no signs of hypertrophy, the augmentation of glucose metabolism compared to control mice might be indicative of deceleration of the normal developmental shift toward FA oxidation, analogously to observed suppression of functional and structural development of the heart. Supporting this, the metabolic shift was not associated with vast transcriptional reprogramming of genes commonly associated with myocardial lipotoxicity (Sletten, Peterson, & Schaffer, 2018). Instead, the only gene with altered expression was Acyl‐CoA thioesterase 1 (*Acot1*) which was increased in HFD hearts (Figure 4d). ACOT1 is a cytosolic protein that catalyzes the hydrolysis of acyl‐CoAs to free FAs and CoA (Hunt et al., 2005). Myocardial expression of ACOT1 has been shown to be increased by HFD (Fujita et al., 2011) and in obese db/db mouse hearts (Yang et al., 2012). Upregulation of ACOT1 is suggested to reduce mitochondrial stress caused by the imbalance between β‐oxidation and TCA cycle during FA overload (Fujita et al., 2011). In line with this idea, ACOT1 overexpression in the heart has been shown to reduce oxidative stress and improve mitochondrial function in db/db mice (Yang et al., 2012). Our data suggest that the HFD induced ACOT1 expression might be the first line of defense against lipotoxicity, as its gene expression is upregulated before any other signs of metabolic remodeling take place during short HFD in adolescent mice.

Based on studies with transgenic and knock‐out mouse models, the pathway downstream from insulin‐like growth factors, consisting of PI3K and Akt is indispensable in both physiological cardiac hypertrophy and developmental growth (Cho, Thorvaldsen, Chu, Feng, & Birnbaum, 2001; DeBosch & Muslin, 2008; DeBosch et al., 2006; McMullen et al., 2003; Shioi et al., 2000; Shiojima et al., 2002). Our results indicate that a short HFD interfered with the activity of InsR‐PI3K‐Akt‐pathway by suppressing the expression of PI3K subunit p110α, Akt1, and phosphorylation of Akt1 (Figure 5). It appears that the pathways mediating physiological growth in conditions with high endogenous activity of PI3K and Akt, such as developmental growth and exercise, are especially sensitive to FAs. Supporting this idea, our data suggest that even relatively short high fat diet interferes with PI3K‐Akt‐pathway restricting the final steps of growth and functional maturation before adulthood. Also other diet compositions leading to obesity and FA accumulation could produce the same outcome, as PI3K inhibition and excess FA intake are strongly linked (Fang et al., 2008; Han et al., 2018; Zhang et al., 2012).

In HFD studies the age of mice, as well as the duration and composition of diet can affect the outcomes drastically. High fat diets on young mice of different ages (7–16 weeks of age) and variety of diet durations (16–44 weeks) have been shown to cause contractile dysfunction and hypertrophy (Abdurrachim et al., 2014; Fang et al., 2008; Wang et al., 2015; Zeng et al., 2015). Comparing to our results, a previous study showed that the same duration of diet with higher fat proportion (60% of kcal from fat) on younger mice (8 weeks) also induced cardiac hypertrophy (Unsold, Bremen, Didie, Hasenfuss, & Schafer, 2015). Furthermore, studies have revealed that HFDs started at different age effect mice very differently, with contradictory results on whether young or old mice are more susceptible to adverse effects of HFD (Aurich et al., 2013; Vercalsteren et al., 2019). Our study was designed to portray the early effects of Western type high fat diet at late adolescence. The mild changes seen here could be the ones that precede the activation of compensating mechanisms and pathologic hypertrophy often associated with longer HFD.

In conclusion, our results show that excessive consumption of FAs during late adolescence suppresses physiological maturation of the heart. These changes are coinciding with diminished activity of the growth promoting PI3K‐Akt‐signalling. Extrapolating these findings from mice to humans in not straight forward since the two species have many dissimilarities in their metabolism, size and aging process. Despite this, organ system, growth regulation, as well as cardiac structure and development are fairly similar (Demetrius, 2005; Wessels & Sedmera, 2003). Moreover PI3K‐Akt‐pathway is a conserved growth regulator throughout all animal kind (Franke, 2008), so it is safe to assume that similar mechanisms might prevail in humans as well.

## CONFLICT OF INTEREST

The authors declare that the research was conducted in the absence of any commercial or financial relationships that could be construed as a potential conflict of interest.

## AUTHOR CONTRIBUTIONS

HH, MM, NN, TT, and PT contributed to conception and design of the experiments and interpretation of results. HH, MM, NN, TT, and AS contributed to collection and analysis of data. PT, HH, and MM contributed to writing the manuscript. All authors approved the final version of the manuscript.
